# The effect of Bafa Wubu of Tai Chi on college students’ anxiety and depression: A randomized, controlled pilot study

**DOI:** 10.3389/fphys.2023.1036010

**Published:** 2023-01-25

**Authors:** Jianwei Zhang, Tianming Gao, Yameng Li, Zhenshao Song, Meize Cui, Qiuyang Wei, Zaihao Chen, Fang Peng, Shaojun Lyu

**Affiliations:** ^1^ College of Physical Education and Sports Science, Beijing Normal University, Beijing, China; ^2^ Student Psychological Counseling and Service Center, Beijing Normal University, Beijing, China; ^3^ Department of PE, Peking University, Beijing, China

**Keywords:** Bafa Wubu of Tai Chi, anxiety, depression, college students’, RS-fMRI

## Abstract

**Objective:** This pilot study aimed to explore the mechanism of the effects of Bafa Wubu of Tai Chi (BWTC) on anxiety and depression in college students using resting-state functional magnetic resonance imaging (RS-fMRI).

**Methods:** Eighteen college students (5 males and 13 females) with anxiety and depression met the study criteria and were randomly divided into an experimental group (aged 24.20 ± 4.07 years) and a control group (aged 22.50 ± 5.95). The experimental group received an eight-week BWTC intervention five times/week for 60 min/session. The control group maintained normal daily life without any exercise intervention. These students were assessed using RS-fMRI scans, the self-rating anxiety scale (SAS), and the self-rating depression scale (SDS). Spearman correlation analysis was used, and statistical significance was defined as a two-sided *p*-value of <0.05.

**Results:** After the intervention, the SAS and SDS scores of the BWTC group significantly reduced (*p* = 0.002; *p* = 0.001). Compared with the control group, the fALFF values of the right middle frontal gyrus, orbital part (Frontal_Mid_Orb_R) (*p* = 0.043), right inferior occipital gyrus (Occipital_Inf_R) (*p* = 0.003), and right middle temporal gyrus of the temporal pole (Temporal_Pole_Mid_R) (*p* = 0.003) in the BWTC group increased significantly; the fALFF values of the left middle frontal gyrus (Frontal_Mid_L) (*p* = 0.001) and right supplementary motor area (Supp_Motor_Area_R) (*p* = 0.010) in BWTC group decreased significantly. The fALFF values of Frontal_Mid_Orb_R were significantly positively correlated with the SDS score (r = 0.852, *p* = 0.015) and the fALFF values of Frontal_Mid_L were significantly negatively correlated with the SAS score (r = −0.797, *p* = 0.032).

**Conclusion:** In this pilot study with college students, BWTC alleviated anxiety and depression, potentially through modulating activity in the Frontal_Mid_L and Frontal_Mid_Orb_R, respectively.

## 1 Introduction

According to the theory of emerging adulthood, college students belong to a unique developmental phase spanning from late adolescence to adulthood (aged 18–29 years) (Arnett, 2000; Jin et al., 2023). College students face a wide range of changes in cognitive function (O’Rourke et al., 2020; Taylor and Snyder, 2021), emotional regulation, and behavior, which are linked to a greater possibility of engaging in risky behaviors e.g., Internet (or smartphone) addiction (Lu et al., 2020), alcohol abuse (Nelson and Padilla-Walker, 2013), alcohol-related sexual behavior (Litt et al., 2020; Persike et al., 2020; Edgerton and Keough, 2021), and developing mental health issues e.g., distorted eating habits, depression, and anxiety (Chi et al., 2020; Lin et al., 2020; Berge et al., 2021; Zhang et al., 2022) due to poor adjustment during this transitional period. Notably, anxiety and depression among college students are the most reported negative emotions considered as risk factors that affect physical (Fuller-Rowell et al., 2021) and mental health (Guastaferro and Bray, 2020; Halliburton et al., 2021), social function (Reed-Fitzke et al., 2021), academic performance (Gillen-O’Neel et al., 2021; Haikalis et al., 2022, and later career success/employment (Masdonati et al., 2022). Previous studies have indicated that 20%–30% of college students report different levels of anxiety and depression (Islam et al., 2020). The two intertwined negative emotions do not simply place a significant financial burden on individuals and their families but also challenge the national healthcare system (Simon et al., 1995). Therefore, there is an urgent need to identify effective strategies to prevent and treat these mental health issues.

Accumulating evidence indicates that non-pharmacological intervention (e.g., cognitive behavioral therapy, mindfulness/mediation, and mindful exercise) has beneficial effects on relieving anxiety and depressive symptoms (Neufeld et al., 2020; Chi et al., 2021; Pardos-Gascón et al., 2021; Pérez-Aranda et al., 2021). Compared to Western psychotherapy, Eastern medicine, especially traditional Chinese medicine (TCM), has received great attention over the past two decades. Within the theory of Chinese medicine, traditional Chinese mind-body exercise (e.g., Tai Chi, Baduanjin, and Wuqingxi) characterized by slow eye-hand and eye-foot coordinative movement, rhythmic deep breathing, and meditative state (Yeung et al., 2018) has become more popular worldwide (Zou et al., 2017; Zou et al., 2018). Notably, these beneficial features of Tai Chi are suitable for vulnerable populations, such as college students suffering from anxiety and depressive symptoms in school settings (Chang et al., 2021). For example, a seminar meta-analysis by Zhang et al. (2019) has concluded that Tai Chi is a cost-effective non-pharmacological approach for relieving anxiety and depressive symptoms among non-clinical individuals across different age groups including college students.

Notably, the potential neurobiological mechanisms underlying the alleviative effects of Tai Chi on depression and anxiety are still not fully understood. With emerging imaging techniques in recent years, exercise scientists have developed new tools to study changes in brain activity, structure, and function due to Tai Chi practice-induced changes (Yue et al., 2020a; Yue et al., 2020b; Chen et al., 2020; Yue et al., 2020c; Yu et al., 2021). Of note, previous imaging studies rarely used resting-state functional magnetic resonance imaging (RS-fMRI), which, as an emerging data acquisition tool, can measure functional connections and local neural activity in the brain in response to changing emotional states (Sparacia et al., 2019). This study used RS-fMRI to explore the effects of Bafa Wubu of Tai Chi (BWTC) on anxiety and depression in college students, and determine whether BWTC can effectively alleviate these negative emotions. This pilot study hypothesized that BWTC could decrease the levels of anxiety and depression in college students and that the neurological mechanism could be examined by fMRI.

## 2 Materials and methods

### 2.1 Participants

A total of 33 college students who reported different levels of anxiety and depression were recruited from the Student Psychological Counseling and Service Center of Beijing Normal University. After evaluation according to the inclusion and exclusion criteria, only 18 college students (five males and 13 females) were retained in the sample. The participants reported an age range of 18–30 years old. They voluntarily agreed to participate in this study and signed informed consent forms. The inclusion criteria were as follows: 1) a self-rating anxiety scale (SAS) (Zung, 1971) score of no less than 50, and 2) a self-rating depression scale (SDS) (Zung et al., 1965) score of no less than 53. The exclusion criteria were as follows: 1) major physical illness or serious mental illness, 2) taking medication, 3) movement disorders and serious joint damage, and 4) presence of metal objects in the body, such as pacemakers.

### 2.2 Study design

This pilot study involved college students. The participants were randomly assigned into two groups (1:1 ratio): the BWTC and control groups. The intervention lasted for 8 weeks. The research process included recruitment, screening, randomization, intervention, and follow-up. This study was approved by the Experimental Ethics Committee of the Department of Psychology of Beijing Normal University and conducted in compliance with the Declaration of Helsinki.

### 2.3 SAS and SDS

SAS was used to test students’ anxiety. SAS was tested for reliability with an intraclass correlation coefficient (ICC) of 0.908 (Zung, 1971). Participants were asked to rate 20 items on a scale of 1–4. The total score for each participant was then multiplied by 1.25. The anxiety score showed a positive correlation with anxiety tendency, and the anxiety of a participant was considered a critical value if his/her score reached 50 (Olatunji et al., 2006).

The SDS was used to test participants’ depression. SDS was tested for reliability with an intraclass correlation coefficient (ICC) of 0.937 (Zung et al., 1965). The main statistical indices, scoring criteria, and statistical methods employed by this scale were the same as those used by the anxiety self-rating scale. The depression score showed a positive correlation with depression tendency, and the depression of a participant was considered a critical value if his/her score reached 53 (Wittkamp et al., 2018).

### 2.4 Sample size calculation

SAS and SDS were set as coprimary outcomes and used for sample size calculation. The sample size was calculated based on the changes in the SAS and SDS between comparison groups with a significance level of 5% and a two-tailed critical region to detect an effect size of Cohen’s d = 0.50, with 80% power using G*Power V.3.1.9.6 software. Post-intervention, the mean scores of SAS and SDS in the control and intervention groups were (43.9 ± 5.6, 32.3 ± 4.5) and (53.6 ± 8.7, 39.2 ± 9.3) (Li et al., 2019), respectively, according to the published literature. Because the sample size calculation of SAS was smaller than that of SDS, the sample size calculation of SDS was selected. This would require 12 participants, increased to 18 to account for the failure to follow-up approximately 30% of participants, with nine participants assigned to each group.

### 2.5 Randomization and blinding

Eighteen participants were randomized into groups using SPSS 21.0 software. The steps were as follows: 1) Encode 18 participants 1–18, 2) set the random number of seeds (0–2000000), 3) generate random numbers (ranged 0–1), 4) sort and group (using the visual discretization method, the number of segmentation points is 1, the width is 50%, and the cut point value is 0.318), and 5) the results of the random grouping were obtained. Blindfolding the participants was difficult because of the characteristics of the intervention measures. Hence, only indicator testers and data analysts were blindfolded.

### 2.6 Intervention plans

The college students in the intervention group received a 1-h BWTC exercise intervention five times a week for eight weeks. The intervention involved 10 min of warm-up activities, 40 min of practice, and 10 min of closing activities. The intervention emphasized not only the postures but also the combination of body, breath, and mind, given that BWTC requires one to “build his/her body,” “convey his/her breaths,” and “use his/her mind” (Lyu, 2018). Participants in the control group went on with their daily lives as usual and did not perform regular physical activities. None of the participants were taking psychotropic or other drugs with similar effects during the study.

### 2.7 Data acquisition

Data were collected at the Magnetic Resonance Laboratory of the Magnetic Resonance Imaging Research Center of Peking University. A Siemens 3tPrisma MRI scanner was used for the data collection. The MRI real radiology department was equipped with an eight-channel phased array head MRI system, which was also used for the acquisition. Functional imaging was performed using gradient echo planar imaging sequences. The scanning parameters obtained included repetition time (TR) = 2,000 ms, echo time (TE) = 30 ms, field of view (FOV) = 220 mm × 220 mm, flip angle (flip angle) = 90°C, matrix = 64 × 64, number of layers = 36, layer thickness = 4 mm, and scan time = 480 s. To mitigate noise from the scanner, each participant was given earplugs before the test. At the beginning of the test, the participants lay flat on the scanner with their heads fixed. They were required to keep their heads and bodies still and remain in an awake and relaxed state throughout the test.

### 2.8 Data processing

The data were analyzed and processed on the Matlab13b platform using the Data Processing Assistant for RS-fMRI software. The data were processed as follows: 1) data format conversion: the data format was converted from Digital Imaging and Communications in Medicine (DICOM) to the Neuroimaging Informatics Technology Initiative (NIfTI); 2) removal: the data for the first 10 time points were removed (the test involved 240 time points, and only 230 time points were used as experimental data); 3) slice timing: the middle of the total number of layers was set; 4) realign: according to the head movement correction curve, participants showing a U-turn movement in the x-, y-, and *z*-axes with translations greater than 1 mm and rotations greater than 1°C were excluded; 5) normalize: the inter-individual differences were reduced to an MNI ERI template through spatial normalization. The voxel resampling was 3 mm × 3 mm × 3 mm; 6) smooth: the Gaussian smoothing kernel with a full width at half maximum (FWHM) of 6 mm was used to smoothen the noise reduction; 7) detrend: the temperature increases due to the work of the machine or the participants were adapted, and the accumulated data collection time showed a linear trend; and 8) calculate the ratio of low-frequency amplitude fraction (fALFF): the root of the power spectrum of the 0.01 Hz–0.08 Hz signal was used to obtain the ALFF value, and the ratio of the amplitude sum in the low-frequency band to the amplitude sum of the whole frequency band was used to obtain the fALFF value.

### 2.9 Statistical analysis

The SPSS 21.0 statistical software was used for the data processing. Continuous variables are described as mean ± standard deviation (SD) for normal distributions or median for non-normal distributions, and categorical variables are described as frequency. The baseline data mainly describe the characteristics of the participants. Two-way repeated measures ANOVA was used to analyze the effects of anxiety and depression before and after the intervention in the two groups and the interaction between them. Statistical parametric mapping (SPM12) software was used for statistical analysis. The fALFF values of different brain regions were extracted and *post hoc* tests were performed. Age, sex, height, and weight were used as covariates, and the Bonferroni correction was used. Paired-sample *t*-tests were used for within-group comparisons, and independent-sample *t*-tests were used for between-group comparisons. Within-group comparisons were performed with Family Wise Error (FWE) correction, and the union of one-sample *t*-test results of the two groups were used as the range of brain functional map comparisons (*p* < 0.05, number of voxels > 20). Comparisons between groups were performed with the FWE correction, and brain regions with *p* < 0.05 and the number of voxels > 20 were defined as statistically different regions. We used xjview and BrainNet Viewer software to present the results. Spearman correlation analysis was used to analyze the correlation between changes in brain area parameters and SAS and SDS scores. Statistical significance was defined as a two-sided *p*-value of <0.05.

## 3 Results

### 3.1 Baseline sample characteristics

Before the intervention, no significant differences were observed between the two groups in terms of age (*p* = 0.483), sex (*p* = 0.736), height (*p* = 0.475), weight (*p* = 0.500), SAS score (*p* = 0.604), or SDS (*p* = 0.490) (Table 1).

**TABLE 1:** Baseline sample characteristics.

Items	BWTC group (*n* = 9)	Control group (*n* = 9)	*p-value*
Age (years)	24.20 ± 4.07	22.50 ± 5.95	0.483
Sex (n,%)	Male 2 (22.2%)	Male 3 (33.3%)	0.736
	Female 7 (77.8%)	Female 6 (66.7%)	
Height (cm)	167.90 ± 5.06	165.63 ± 8.07	0.475
Weight (kg)	61.78 ± 11.78	57.50 ± 11.46	0.500
SAS (score)	56.94 ± 9.23	54.58 ± 9.70	0.604
SDS (score)	61.80 ± 10.53	57.50 ± 14.92	0.490

Note: SAS: self-rating anxiety scale; SDS: self-rating depression scale.

### 3.2 Comparison of SAS and SDS scores in the two groups

Compared to the change in SAS, there was no interaction between group and time (F = 1.660, *p* = 0.245). The results showed that the change in SAS scores had a main effect of time (F = 26.975, *p* = 0.002). Compared with before intervention, SAS in the BWTC group was significantly reduced after intervention (56.94 ± 9.23 vs. 41.60 ± 8.85, *p* < 0.01), while there was no significant difference in the control group (*p* > 0.05). Compared to the change in SDS, there was no interaction between group and time (F = 2.235, *p* = 0.186). The results showed that the change in SDS had a main effect of time (F = 41.324, *p* = 0.001). Compared with before intervention, SDS in the BWTC group was significantly reduced after intervention (69.29 ± 12.16 vs. 43.39 ± 10.17, *p* < 0.01), while there was no significant difference in the control group (*p* > 0.05). The results are presented in Tables 2, 3.

**TABLE 2:** Two-way repeated measures ANOVA of SAS and SDS.

Indicators	Group		Time		Group × time	
SAS	F	*p*	F	*p*	F	*p*
	0.159	0.704	26.975	0.002	1.660	0.245
SDS	F	*p*	F	*p*	F	*p*
	0.305	0.601	41.324	0.001	2.235	0.186

Note: SAS: self-rating anxiety scale; SDS: self-rating depression scale.

**TABLE 3:** Comparison of the SAS and SDS scores of the two groups (*_x* ± s).

Indicators	Time	N	BWTC group	Control group
SAS	Before intervention	9	56.94 ± 9.23	54.58 ± 9.70
	After intervention	9	41.60 ± 8.85**	47.52 ± 8.72
SDS	Before intervention	9	69.29 ± 12.16	57.50 ± 11.32
	After intervention	9	43.39 ± 10.17**	51.80 ± 12.16

Note: SAS: self-rating anxiety scale; SDS: self-rating depression scale. Compared with before intervention, **p < 0.01.

### 3.3 Comparison of RS-fMRI between the two groups after the intervention

**FIGURE 1:**
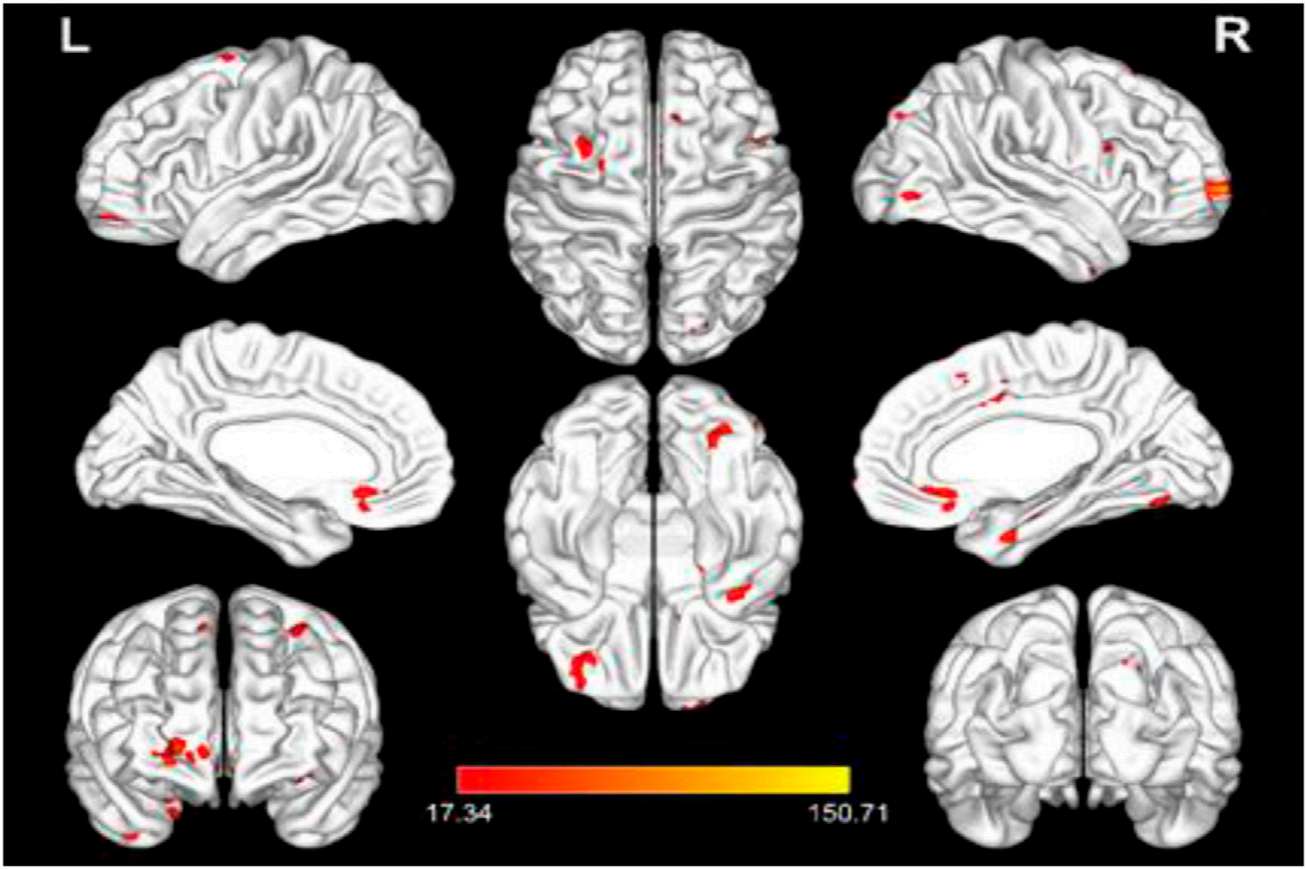
3D plot of the significant interaction of fALFF values. Note: The red bars represent those brain regions with significant interactions, and the statistical threshold was set to *p* < 0.001 and the number of voxels was > 20. L: left; R: right; and fALFF: low-frequency amplitude fraction.

**FIGURE 2:**
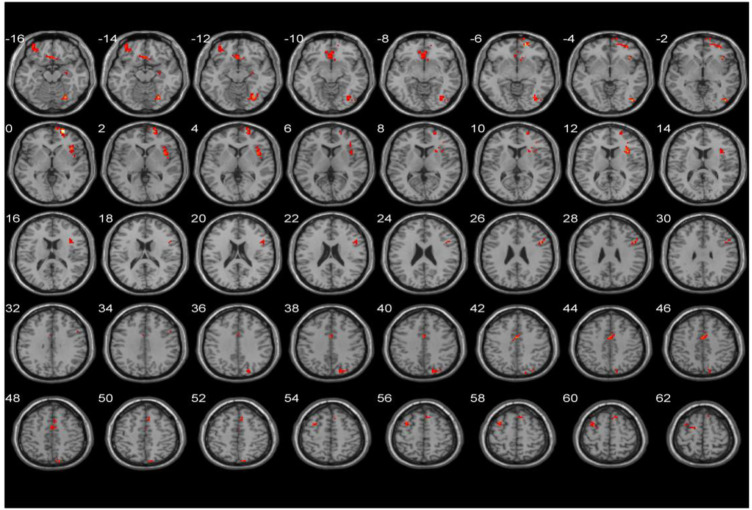
Significant plane diagram of the interaction effect of fALFF values. Note: The color bar represents the brain area with significant interaction, the statistical threshold was set to *p* < 0.001, and the number of voxels was > 20. fALFF: low-frequency amplitude fraction.

After the intervention, the interaction between the fALFF values in the right middle frontal gyrus, orbital part (Frontal_Mid_Orb_R), left middle frontal gyrus (Frontal_Mid_L), right superior frontal gyrus (Frontal_Sup_R), right inferior occipital gyrus (Occipital_Inf_R), right superior occipital gyrus (Occipital_Sup_R), right supplementary motor area (Supp_Motor_Area_R), left inferior temporal gyrus (Temporal_Inf_L), and right middle temporal gyrus of the temporal pole (Temporal_Pole_Mid_R) of the two groups changed significantly. The results are shown in Table 4; Figures 1, 2.

**TABLE 4:** Comparison of the fALFF values between the two groups after the intervention.

	Brain areas	Number of voxels	MNI			F
			X	Y	Z	
1	Frontal_Mid_Orb_R	64	0	27	−12	50.2123
2	Frontal_Mid_L	40	−30	6	60	51.55
3	Frontal_Sup_R	76	24	63	0	150.71
4	Insula_R	76	36	15	12	73.68
5	Occipital_Inf_R	59	45	−78	−3	84.61
6	Occipital_Sup_R	31	21	−84	39	43.81
7	Supp_Motor_Area_R	20	9	21	51	39.23
8	Temporal_Inf_L	22	−39	−3	−30	66.21
9	Temporal_Pole_Mid_R	79	21	−3	−30	67.23

Note: Results of the independent sample *t*-test, *p* < 0.05, number of voxels > 20, Family wise error correction. X: left and right; Y: front and back; Z: up and down; and F: the t-value of the Peak point, the one with the most significant difference in this brain region. fALFF: low-frequency amplitude fraction. The MNI, coordinate values are based on the Montreal Institute standard template space. Frontal_Mid_Orb_R: right middle frontal gyrus, orbital part; Frontal_Mid_L: left middle frontal gyrus; Frontal_Sup_R: right superior frontal gyrus; Insula_R: right insula; Occipital_Inf_R: right inferior occipital gyrus; Occipital_Sup_R: right superior occipital gyrus; Supp_Motor_Area_R: right supplementary motor area; Temporal_Inf_L: left inferior temporal gyrus; Temporal_Pole_Mid_R: right middle temporal gyrus of the temporal pole.

**FIGURE 3:**
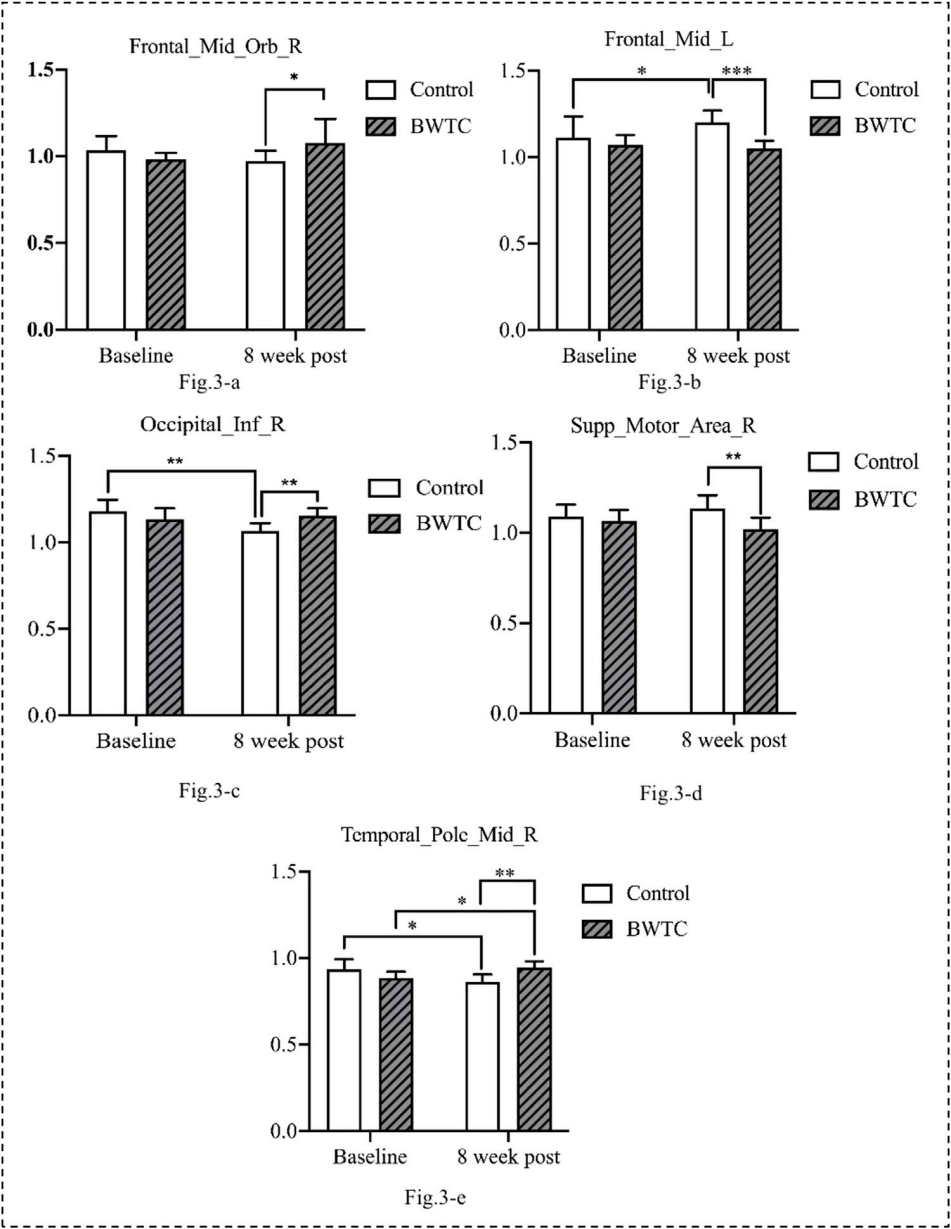
The differences between the groups before and after the intervention in the same brain region.

To further analyze the differences between the groups before and after the intervention in the same brain region, the fALFF values of different brain regions were extracted for *post hoc* analysis. After the intervention, compared with the control group, the fALFF values of the Frontal_Mid_Orb_R (*p* = 0.043), Occipital_Inf_R (*p* = 0.003), and Temporal_Pole_Mid_R (*p* = 0.003) in the BWTC group increased significantly, indicating that the local activity of the corresponding brain regions was enhanced, while the fALFF values of Frontal_Mid_L (*p* = 0.001) and Supp_Motor_Area_R (*p* = 0.010) in the BWTC group decreased significantly, indicating that the local activity of the corresponding brain regions was weakened. The results are shown in Figure 3.

To further explore the potential correlation between anxiety and depression and local brain activity after the intervention, the correlation coefficients of fALFF values and SAS-SDS scores were calculated using Spearman correlation analysis. The fALFF values of Frontal_Mid_Orb_R were significantly positively correlated with the SDS score (r = 0.852, *p* = 0.015) and the fALFF values of Frontal_Mid_L were significantly negatively correlated with the SAS score (r = −0.797, *p* = 0.032) (Table 5).

**TABLE 5:** Correlation between SAS-SDS and local brain activity after intervention.

Brain areas	SAS		SDS	
	r	*p*	r	*p*
Frontal_Mid_Orb_R	0.357	0.432	0.852	0.015
Frontal_Mid_L	−0.797	0.032	−0.445	0.317
Frontal_Sup_R	−0.214	0.645	−0.037	0.937
Insula_R	−0.250	0.589	0.482	0.274
Occipital_Inf_R	0.107	0.819	−0.148	0.751
Occipital_Sup_R	−0.179	0.702	−0.074	0.875
Supp_Motor_Area_R	−0.429	0.337	0.371	0.413
Temporal_Inf_L	0.214	0.645	0.036	0.757
Temporal_Pole_Mid_R	−0.357	0.432	−0.057	0.837

Note: SAS: self-rating anxiety scale; SDS: self-rating depression scale. Frontal_Mid_Orb_R: right middle frontal gyrus, orbital part; Frontal_Mid_L: left middle frontal gyrus; Frontal_Sup_R: right superior frontal gyrus; Insula_R: right insula; Occipital_Inf_R: right inferior occipital gyrus; Occipital_Sup_R: right superior occipital gyrus; Supp_Motor_Area_R: right supplementary motor area; Temporal_Inf_L: left inferior temporal gyrus; Temporal_Pole_Mid_R: right middle temporal gyrus of the temporal pole.

## 4 Discussion

To the best of our knowledge, this study is the first to explore the mechanism underlying the effects of BWTC on anxiety and depression in college students using RS-fMRI. After eight weeks of BWTC, the participants in the BWTC group showed significant improvements in anxiety and depression, whereas those in the control group showed no significant changes. The RS-fMRI results showed that, compared with the control group, the Frontal_Mid_Orb_R, Occipital_Inf_R, and Temporal_Pole_Mid_R in the BWTC group were enhanced, and Frontal_Mid_L and Supp_Motor_Area_R in the BWTC group were weakened after the intervention. This study further explored the potential correlation between anxiety, depression, and local brain activity after the intervention. Frontal_Mid_Orb_R was significantly positively correlated with depression and Frontal_Mid_L had a significant negative correlation with anxiety.

Yang et al. (2016) found that depression and anxiety can be alleviated by modulating the functional connectivity between the interior and insular cortices of the dorsal cingulate gyrus, suggesting that the regulation of anxiety and depression is closely related to the functional connectivity of the brain. In other words, anxiety and depression can be alleviated by improving the functional connectivity of the local brain regions. Gold et al. (2015) identified the insula, thalamus, striatum, cingulate cortex, and amygdala as brain regions associated with anxiety, whereas Etkin et al. (2011) identified the insula, paracingulate cortex, and medial prefrontal cortex as brain regions associated with emotional engagement and processing, especially fear and anxiety. The insula, prefrontal cortex, anterior cingulate cortex, and amygdala play important roles in the development, processing, and regulation of depression and anxiety. Therefore, BWTC exercise improves anxiety and depression mainly by modulating the functional connectivity and neural activity of related brain regions. This study found that BWTC may relieve anxiety and depression by regulating neural activity in the Frontal_Mid_L and Frontal_Mid_Orb_R, respectively.

This study also analyzed the reasons why BWTC effectively alleviated anxiety and depression among college students. During practice, participants in the BWTC group were required to cooperate closely with their minds. Previous studies have shown that the mind completes information transmission through bioelectricity accompanied by neural and humoral regulation, generates neuronal excitement, and places the cerebral cortex in a special state of excitation, thereby regulating psychological and physiological activities (Ray, 2004; Hassan et al., 2019; Tang et al., 2019). This study attributes this effect to the emphasis of BWTC on the oneness of the body and mind, which stimulated the brain regions of the participants to a certain extent. Such stimulation generates bioelectrical signals that lead to excitation and biochemical reactions, such as neurotransmitters and hormones, and activates key brain regions for emotion regulation, such as Frontal_Mid_L and Frontal_Mid_Orb_R. The brain network connections and mechanisms of these brain regions are optimized, thereby alleviating anxiety and depression in college students. BWTC is often accompanied by relaxing and soothing music, which can stimulate the auditory nervous system, relax tense nerves, and subsequently alleviate anxiety and depression (Petrovsky et al., 2015). A meta-analysis of 44 original studies by Song found that aerobic exercise may have a better effect on anxiety and stress, whereas traditional Chinese exercise may have a better effect on stress. This study found that BWTC might improve anxiety and depression.

This pilot study had several limitations. First, additional physical activities of the participants throughout the study could not be easily monitored. Second, the sample size was relatively small and the intervention was delivered over a relatively short period. Future studies should employ larger samples and longer in-depth exercise durations to confirm the findings of this study.

## 5 Conclusion

In this pilot study with college students, BWTC had the potential to alleviate anxiety and depression in college students by regulating neural activity in Frontal_Mid_L and Frontal_Mid_Orb_R, respectively.

## Data Availability

The raw data supporting the conclusion of this article will be made available by the authors, without undue reservation.
